# Nanomedicine for cancer patient‐centered care

**DOI:** 10.1002/mco2.767

**Published:** 2024-10-20

**Authors:** Carlo Sorrentino, Stefania Livia Ciummo, Cristiano Fieni, Emma Di Carlo

**Affiliations:** ^1^ Department of Medicine and Sciences of Aging “G. d'Annunzio” University” of Chieti‐Pescara Chieti Italy; ^2^ Anatomic Pathology and Immuno‐Oncology Unit, Center for Advanced Studies and Technology (CAST) “G. d'Annunzio” University of Chieti‐Pescara Chieti Italy

**Keywords:** drug delivery systems, nanomedicine, nanotechnology, personalized cancer treatments, precision oncology, targeted therapy

## Abstract

Cancer is a leading cause of morbidity and mortality worldwide, and an increase in incidence is estimated in the next future, due to population aging, which requires the development of highly tolerable and low‐toxicity cancer treatment strategies. The use of nanotechnology to tailor treatments according to the genetic and immunophenotypic characteristics of a patient's tumor, and to allow its targeted release, can meet this need, improving the efficacy of treatment and minimizing side effects. Nanomedicine‐based approach for the diagnosis and treatment of cancer is a rapidly evolving field. Several nanoformulations are currently in clinical trials, and some have been approved and marketed. However, their large‐scale production and use are still hindered by an in‐depth debate involving ethics, intellectual property, safety and health concerns, technical issues, and costs. Here, we survey the key approaches, with specific reference to organ‐on chip technology, and cutting‐edge tools, such as CRISPR/Cas9 genome editing, through which nanosystems can meet the needs for personalized diagnostics and therapy in cancer patients. An update is provided on the nanopharmaceuticals approved and marketed for cancer therapy and those currently undergoing clinical trials. Finally, we discuss the emerging avenues in the field and the challenges to be overcome for the transfer of nano‐based precision oncology into clinical daily life.

## Graphical abstract

Nanomedicine can satisfy the need for developing precision oncology, promote modern early diagnostic systems, monitoring the therapy based on the response of the individual patient, and provide precision antitumor immunotherapy and gene therapy, which can be developed with sophisticated experiments such as those made with organ‐on‐chip technology.

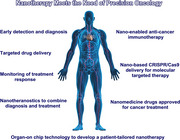

## 1 INTRODUCTION

According to WHO and other key statistics, cancer is a leading cause of death worldwide, accounting for nearly 10 million deaths in 2020.^1^ The development of effective therapeutic strategies tailored to the genetic profile and anatomo‐clinical characteristics of the patient's tumor has become urgent.

The concept of nanotherapy can be traced back to the 1959 lecture by physicist Richard Feynman, titled “There's Plenty of Room at the Bottom” where he discussed the possibilities of manipulating individual atoms and molecules.^2^, ^3^ The term “nanotechnology” was used for the first time in a book by K. Eric Drexler, dated 1986, entitled “Engines of Creation: The Coming Era of Nanotechnology.” However, it was not until the early 21st century that the field of nanomedicine started gaining momentum.^5^ Nanobiotechnology has made huge advancements in the field of oncology for its ability in achieving precise and controlled drug release, increasing drug solubility, and reducing adverse effects, thereby improving the precision and effectiveness of medical treatments. Despite advances in cancer research and therapy development, treatment failure due to tumor heterogeneity, development of drug resistance, and systemic toxicities still represent major obstacles for an effective cancer treatment. Nanoscale delivery systems may provide suitable vehicles for antineoplastic and immunoregulatory agents because of their potential for targeting and multifunctionality.^6^ Their versatility has extended their use to the field of diagnostics and noninvasive molecular targeted imaging^7^, ^8^ enabled for the detection of cancer biomarkers and for therapy response monitoring, which can help guide the selection of the most appropriate treatment, tailored to the patient's tumor, and foster the development of precision oncology. The goal of precision oncology is to tailor medical care to the individual characteristics of each patient and their tumor using information regarding a patient's genetic makeup, molecular profile, and other specific features of the cancer to achieve accurate diagnosis and treatment decision‐making.^9^, ^10^ It represents a paradigm shift from the one‐size‐fits‐all approach to cancer treatment toward more personalized therapies based on the unique characteristics of each patient's cancer. The roots of precision oncology can be traced back to advancements in genomics and molecular biology.

The milestones and key factors that contributed to the birth and development of precision oncology, are the following. (a) *Human Genome Project*. The completion of the Human Genome Project in 2003 was a groundbreaking achievement that identified and mapped all the genes in the human genome and laid the foundation for understanding the genetic basis of diseases, including cancer.^11^ (b) *Advancements in sequencing technologies*. Technological advancements in DNA sequencing, such as next‐generation sequencing, has allowed researchers to efficiently and cost‐effectively sequence large amounts of genetic information. This has made it possible to analyze the genomic makeup of individual tumors more comprehensively.^12^ (c) *Cancer Genome Atlas (TCGA)*. Launched in 2005, the Cancer Genome Atlas project aimed to catalog and characterize genomic changes in various cancer types. TCGA provided a vast amount of genomic data, enabling researchers to identify specific genetic alterations associated with different types of cancer.^13^, ^14^ (d) *Identification of driver mutations*. Researchers have identified specific genetic mutations, known as driver mutations, that play a crucial role in the development and progression of cancer. Targeting driver mutations, such as the EGFR mutation in non‐small cell lung cancer, the KRAS mutation in colorectal cancer, the BRAF mutation in melanoma, the FLT3 mutation in acute myeloid leukemia (AML), human epidermal growth factor receptor 2 (HER2) amplification in breast cancer, has become a key strategy in precision oncology.^15^ (e) *Biomarker discovery*. The identification of biomarkers, measurable indicators of a biological state or condition, has allowed for a better categorization of tumors and more accurate predictions of treatment response. Biomarkers serve as tools for the selection of appropriate therapies based on an individual patients’ characteristics. Integration of omics data stemming from genomic, transcriptomic, epigenetic, proteomic, and metabolomic datasets generated from patient cohorts can lead to the discovery of new potential cancer biomarkers.^16^ (f) *Drug development and targeted therapies*. The discovery of specific genetic mutations has led to the development of targeted therapies designed to inhibit or block the effects of these mutations. Drugs like imatinib (Gleevec) and trastuzumab (Herceptin) were among the first successful targeted therapies.^17^ (g) *Liquid biopsies*. The development of liquid biopsies, which involve analyzing circulating tumor DNA in blood samples, has provided a noninvasive method for monitoring tumor dynamics and detecting genetic changes over time.^18^ (h) *Advancements in immunotherapy*. Immunotherapy, particularly immune checkpoint inhibitors (ICIs), CAR T‐cell therapy, and cancer vaccines, has revolutionized cancer treatment and fits well with the concept of precision medicine as it is based on an individual's immune response.^19^, ^20^

The growing use of artificial intelligence (AI) systems in biomedical research has allowed both the design and production of more sophisticated nanostructures and the analysis of multiple parameters that contribute to precision medicine, using deep learning approaches.^21^, ^22^ AI can speed up advanced manufacturing of nanosystems for personalized cancer therapy and diagnosis and shape the future of healthcare.

## 2 HOW NANOTHERAPY MEETS THE NEED OF PRECISION ONCOLOGY

Nanomedicine holds promise for personalized oncology, tailoring treatments to individual patients’ profiles. Nanoformulation of drugs is one strategy to deliver pharmaceutical agents more precisely to the targeted tissue and reduce the overall dose and potentially toxic side effects.^23^ Nanosized formulations, in comparison with conventional microsized formulations, lead to an increased active concentration and bioavailability.^24^ Nanotechnology facilitates the development of personalized and targeted therapies based on the unique genetic and molecular profile of an individual's tumor. AI can be integrated into nanodevices to monitor physiological parameters, to respond to changes, and to adjust treatment accordingly. This closed‐loop system can improve therapeutic outcomes by adapting to the patient's evolving condition.^21^, ^25^ The impact areas of nanotechnology in precision oncology include the following.

### 2.1 Nanobiosensors for early detection and diagnosis

Nanotechnology enables the development of highly sensitive biosensors for the early detection of single or multiple cancer biomarkers, circulating tumor cells (CTCs), or extracellular vesicles secreted by the tumor, in real samples and complex matrices, with high sensitivity and specificity.^26^ Biosensors can detect molecular changes associated with cancer at a very early stage, facilitating early diagnosis and intervention. Circulating biomarkers, such as nucleic acids and proteins can be detected and quantified by nanobiosensors, such as bio‐barcodes, quantum dots (QDs), metal nanoparticles, and carbon‐based nanosensors,^27^, ^28^, ^29^ which convert a biological entity into an electrical signal. Their versatility has been demonstrated in different applications aiming to detect and quantify cancer biomarkers, at low concentrations with high sensitivity and specificity, in real biological samples.

### 2.2 Nano‐based imaging techniques

Nanoparticles can be engineered to carry imaging agents and be used for both anatomic and molecular imaging, allowing for more accurate and specific imaging of tumors.^30^ Compared with conventional contrast agents, nanoparticles can be functionalized with antibodies (Abs) and provide better imaging of target tissues. This enhances the precision of diagnostic techniques such as magnetic resonance imaging (MRI), computed tomography (CT), and positron emission tomography (PET). Inorganic nanomaterials containing metals such as gold, silver, or platinum and magnetic nanoparticles can be used as diagnostic tools for tissue imaging,^31^ although concerns about their long retention in the body and long‐term toxicity (of the MRI or CT contrast‐generating metals or halogens loaded in them) still hamper their use in the clinical practice.

### 2.3 Nanocarriers for targeted drug delivery

Nanoparticles can be designed to carry and deliver drugs directly into cancer cells, minimizing damage to healthy tissue. Nowadays, the delivery of chemotherapeutic drugs is considered the main application of nanocarriers. Nanoparticles can improve chemotherapy solubility and bioavailability, ensuring better distribution in the body. Targeted drug delivery may be achieved by functionalizing nanoparticles with ligands, such as folate ligands,^32^ or Abs, such as anti‐epidermal growth factor receptor (EGFR),^33^, ^34^ that specifically recognize and bind to the receptors on target cells. Paclitaxel or doxorubicin (DOX) are loaded into functionalized gold nanoparticles (GNPs). PEGylation (polyethylene glycol) improves the nanoparticles’ biocompatibility, stability, and circulation time in the bloodstream by reducing immune system recognition and clearance. Conjugation of folate to PEGylated nanoparticles allows binding to the folate receptors, which are usually overexpressed on the surface of cancer cells, providing a specific target for drug delivery. Since the first clinical approval of Doxil® in 1995, lipid‐based nanoparticles remain the most prevalent class of nanopharmaceuticals on the market or in clinical trials.^35^ In addition to acquired immune deficiency syndrome (AIDS)‐related Kaposi's sarcoma (1995), this DOX‐loaded PEGylated liposome (Doxil®) was United States Food and Drug Administration (US FDA) approved in recurrent ovarian cancer (1998), metastatic breast cancer (2003), and multiple myeloma (2007)^36^; hence, it was a real breakthrough in cancer nanomedicine and lipid‐based drug delivery systems (DDSs).

Recent advances in nanoparticle‐based anticancer treatment have led to the development of nanorobotic DDSs, including surgical and cellular repair nanorobots, able to work at cellular levels with nanoscale precision. Nanorobots are mainly made of carbon, due to its inertness, high thermal conductivity, and strength, and are equipped with external diamond coating, to elude host immune system attack. DNA nanorobots can recognize different types of cancer cells^37^ and, due to computer‐controlled distribution, they can release drugs in a very precise and regulated manner, ensuring speed and efficacy of the treatment. Lowering production costs and implementing research to improve their resistance to immune attack could accelerate their transition to clinical use.^38^, ^39^

### 2.4 Nanoplatforms for monitoring treatment response

- *Nanoparticle‐based sensors*. Nanoscale sensors can be used to monitor treatment response in real‐time. This provides valuable information about the effectiveness of therapy, allowing for timely adjustments and improvements in patient outcomes. Nanobiosensors can be used for detection of tumor biomarkers, CTCs, cell‐free DNA, or exosomes in body fluids like blood or urine.^40^ Nanobiosensors can facilitate early detection of cancer relapse,^41^ enabling timely intervention.
- *Nanotechnology‐assisted microfluidic platforms for CTC isolation and analysis*. This noninvasive approach allows the monitoring of disease progression and of treatment response through liquid biopsies.^42^ Further research, validation, and regulatory approval are required for their widespread clinical implementation.
- *Predictive modeling and computational approaches*. The integration of nanotechnology with computational oncology models allows for predictive modeling of drug responses and treatment outcomes.^43^ Mathematical modeling can be used to probe the pharmacokinetics and pharmacodynamics relationships of the available anticancer nanoformulations and to improve treatment. This can aid in optimizing treatment plans for individual patients.^44^

### 2.5 Nanotheranostics to combine imaging modalities and treatment

Theranostic nanoparticles serve diagnostic and therapeutic functions and monitoring of the therapeutic response offering a comprehensive approach to cancer treatment and an effective tool for personalized medicine.^45^ They can be used for noninvasive imaging of tumors (by using MRI, near‐infrared [NIR] fluorescence, photoacoustic, or ultrasound imaging), for targeting image biomarkers and for delivery of therapeutic agents (such as chemotherapeutics, X‐rays, hyperthermia, or free radicals) simultaneously. Several types of nanocarriers have been developed so far for nanotheranostics,^46^, ^47^ such as: (a) dendrimers, which are highly branched tree‐like macromolecules, arranged in a central core of interior branching units, and numerous external terminal functional groups that can be conjugated with therapeutic agents, including small molecule drugs, DNA, small interfering RNA (siRNA), or Abs, which can be protected from enzymatic degradation and delivered into cancer cells. Dendrimers can be engineered to carry multiple imaging agents, enabling multimodal imaging (e.g., combining MRI, PET, and fluorescence imaging) for more comprehensive tumor diagnostics^48^; (b) micelles, which are self‐assembled colloidal nanostructures formed by amphiphilic molecules (e.g., block copolymers). They typically have a hydrophobic core that can encapsulate hydrophobic chemotherapeutic drugs, improving their solubility and bioavailability, and a hydrophilic shell that stabilizes the micelle in aqueous environments. The core–shell structure allows for the efficient loading of drugs, and the micelles can be designed to release the drug in response to specific triggers, such as pH or temperature changes in the tumor microenvironment (TME). Micelles can be functionalized with ligands for specific cancer cell targeting and can be loaded with imaging agents, such as fluorescent dyes, MRI contrast agents, or radionuclides, allowing them to be used for diagnostic imaging.^49^ Like dendrimers, micelles can be engineered for multimodal imaging by incorporating different types of imaging agents; (c) polymer–drug conjugates, which consist of a therapeutic drug covalently linked to a polymer backbone that can protect the drug from degradation in the bloodstream and release it in response to specific triggers (e.g., pH, enzymes) in the TME. These conjugates can be functionalized with targeting ligands to enhance the selective delivery of the drug to cancer cells^50^ and can also carry imaging agents, such as fluorescent dyes, radionuclides, or MRI contrast agents, for tumor imaging.^51^ Like dendrimers and micelles, polymer conjugates can be designed for multimodal imaging; (d) carbon nanotubes (CNTs),^52^, ^53^ which can be used for drug delivery and photothermal ablation, are characterized by a high surface area, which allows for efficient drug loading. Upon exposure to NIR light, they generate heat that can selectively kill cancer cells. CNTs enhance contrast in various imaging modalities, including MRI and optical imaging.^54^, ^55^ Their unique electronic properties also make them ideal for biosensing applications; (e) liposomes can encapsulate chemotherapeutic drugs, protecting them from degradation and allow for the controlled release of the drug at the tumor site. Liposomes can be engineered to carry imaging agents, such as fluorescent dyes or radiolabels, enabling noninvasive tracking of the drug delivery process through fluorescence imaging or PET^56^; (f) solid lipid nanoparticles (SLNs), composed of a solid lipid core, which provides a stable matrix for drug encapsulation, and stabilized by a surfactant or polymer shell, which can encapsulate both hydrophobic and hydrophilic therapeutic agents, can be functionalized with targeting ligands, imaging agents, or surface coatings to improve circulation time and targeting specificity.^57^ For diagnostic purpose, SLNs can incorporate fluorescent dyes for optical imaging or can be loaded with MRI contrast agents, such as gadolinium or iron oxide nanoparticles. SLNs can also be labelled with radionuclides for PET or single‐photon emission computed tomography imaging, enabling the tracking of the nanoparticles and the assessment of their biodistribution^58^, ^59^; (g) GNPs can be used in photothermal therapy, in which they absorb and then convert NIR light into heat, selectively destroying cancer cells. They can also be conjugated with drugs, Abs, or targeting ligands for precise drug delivery. GNPs enhance imaging techniques like CT and surface‐enhanced Raman spectroscopy. Their high electron density makes them excellent contrast agents for X‐ray‐based imaging methods^60^, ^61^, ^62^; (h) magnetic nanoparticles, made of iron oxide (Fe_3_O_4_ or γ‐Fe_2_O_3_), cobalt, or other magnetic materials, are primarily used in magnetic hyperthermia, where they generate localized heat upon exposure to an alternating magnetic field, leading to the thermal ablation of cancer cells. They can also be functionalized to carry chemotherapeutic agents for targeted delivery. These nanoparticles are widely used as contrast agents in MRI due to their superparamagnetic properties, which improve the quality of MRI scans and help in early cancer detection^63^, ^64^; (i) silica nanoparticles, made of silicon dioxide (SiO_2_). Due to their high surface area and porous structure, mesoporous silica nanoparticles (MSNPs) are an ideal tool for the loading and delivery of drugs, proteins, or genes, which can be released in response to specific stimuli like pH or temperature changes in the TME. Silica nanoparticles can be doped with imaging agents like QDs or metal ions, enabling them to be used for optical imaging, fluorescence imaging, or even multimodal imaging when combined with other techniques like MRI or PET^65^; (j) QDs, made of semiconductor materials like cadmium selenide, cadmium telluride, or indium phosphide. QDs can be used for drug delivery and targeted therapy when conjugated with specific ligands or Abs.^66^, ^67^ Their small size and tunable surface chemistry allow for precise targeting of cancer cells. Their superior fluorescence properties, including high brightness and photostability, makes them ideal for imaging applications, such as fluorescence microscopy, which is used to visualize cancer cells and track the distribution of therapeutic agents in vivo.

In summary, nanotechnology plays a crucial role in advancing precision oncology by enabling early detection, accurate imaging, targeted drug delivery, and personalized treatment, which increase efficacy of therapies and minimize side effects, ultimately improving patient outcomes.

## 3 NANO‐ENABLED ANTICANCER IMMUNOTHERAPY

Immunotherapy has revolutionized the treatment of various advanced cancers, exploiting different approaches such as checkpoint inhibitors, lymphocyte‐promoting cytokines, engineered T cells, and cancer vaccines.

However, a key challenge in the widespread implementation of cancer immunotherapy is the precise regulation of the immune system, as these treatments can lead to serious adverse effects including autoimmunity and nonspecific inflammation. Nanoparticles can improve the delivery of immunotherapeutic agents and help to tailor anticancer immunotherapy according to the genetic make‐up, immunophenotype, and immune cell context of the patient's tumor. Advances in immunophenotyping, by using multiplexed tissue imaging platforms, and associated digital pathology, can help define immunoscores and immunograms essential for personalized nano‐based immunological targeting.^68^, ^69^

**FIGURE 1:**
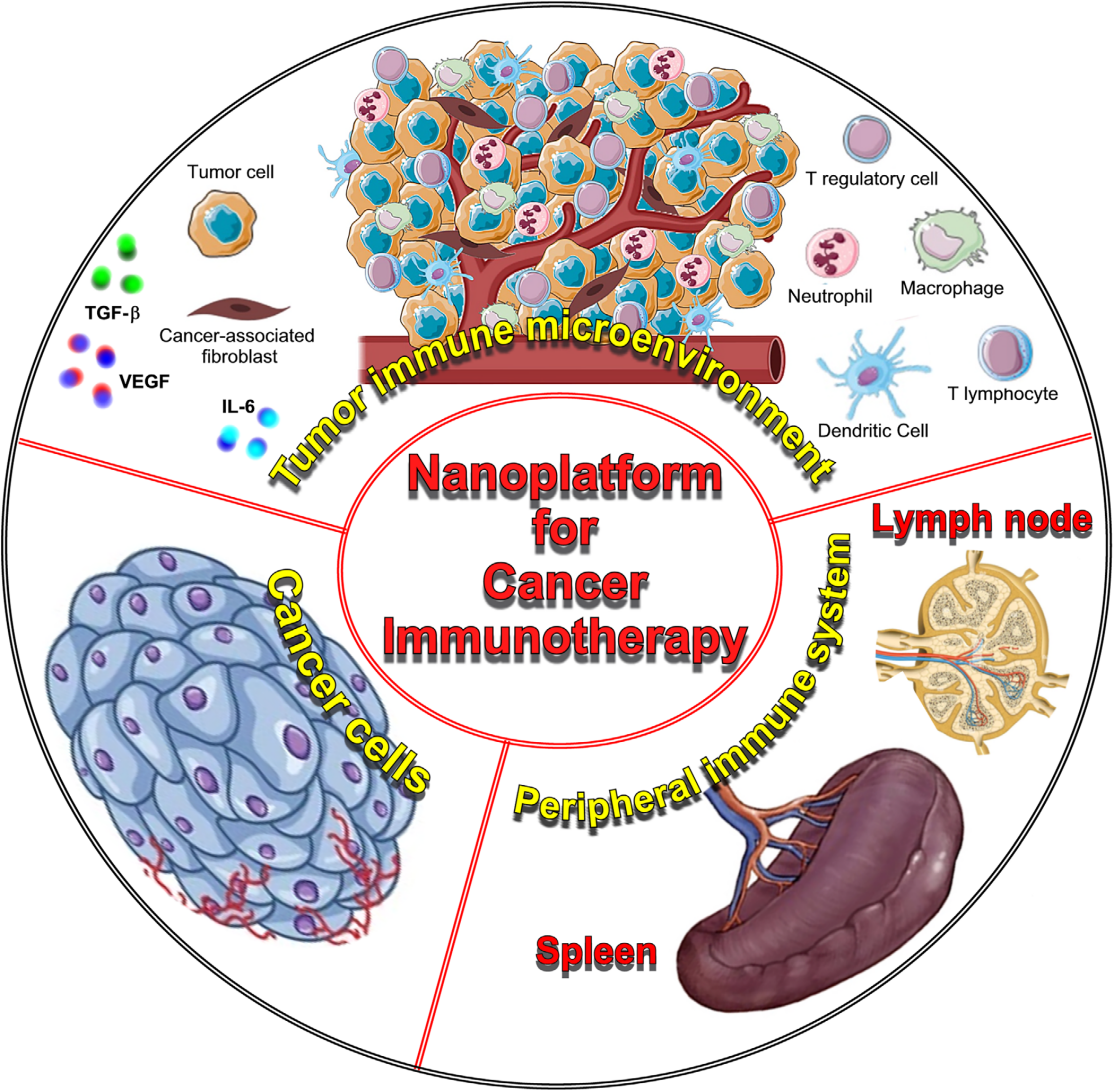
Graphic representation of the three main approaches of nanoimmunotherapy to precision oncology, which consist in: (1) targeting cancer cells (left compartment), which aim to induce immunogenic cell death, thereby triggering the release of tumor antigens and danger‐associated molecular patterns; (2) targeting the tumor microenvironment (top compartment) and re‐educate immunosuppressive cells, such as protumorigenic M2 macrophages, N2 neutrophils, or T regulatory cells, or inhibit the production of immunosuppressive/inflammatory mediators, such as TGF‐β, IL‐6, or VEGF or targeting cancer‐associated fibroblasts to reverse drug resistance, or enhance cytotoxic T lymphocyte and natural killer cell infiltration; (3) targeting the peripheral immune system (right compartment), to enhance tumor‐antigen presentation to dendritic cells, and cytotoxic T cell activation and proliferation in secondary lymphoid organs, thereby promoting anticancer immunity. Created using Microsoft PhotoDraw (version 2.0.0.0822).

Nano‐immunotherapy can be realized via three different approaches,^70^ which use nanomedicines (Figure 1): (1) to target cancer cells,^71^ which aim to induce immunogenic cell death (ICD), thereby triggering the release of tumor antigens and danger‐associated molecular patterns, that function as adjuvants to activate antigen‐presenting cells (APCs) to take up, process, and present the former, thereby promoting the generation of CD8^+^ cytotoxic T cells; (2) to target the tumor immune microenvironment and re‐educate immunosuppressive cells, such as protumorigenic M2‐like tumor‐associated macrophages or myeloid‐derived suppressor cells, or inhibit the production of immunosuppressive/inflammatory mediators, such as TGF‐β, IL‐6, or VEGF, or targeting cancer‐associated fibroblasts to improve the efficacy of treatments and reverse drug resistance, or subvert extracellular matrix composition and reshape its architecture to enhance T cell infiltration, and prevent cancer cell invasion and metastasis^72^, ^73^; (3) to target the peripheral immune system, which aim is to enhance antigen presentation and cytotoxic T cell production in secondary lymphoid organs (for instance, by nanoparticle‐mediated delivery of tumor‐associated antigens or adjuvants directly to dendritic cells [DCs]),^74^ as well as to engineer and strengthen peripheral effector immune cell populations (for instance, by nanoparticle‐mediated delivery of costimulatory molecules, that strengthen T cell activation, or checkpoint inhibitors, such as anti‐CTLA‐4 or anti‐PD‐1, to reverse T cell exhaustion),^75^ thereby promoting anticancer immunity.^76^

**FIGURE 2:**
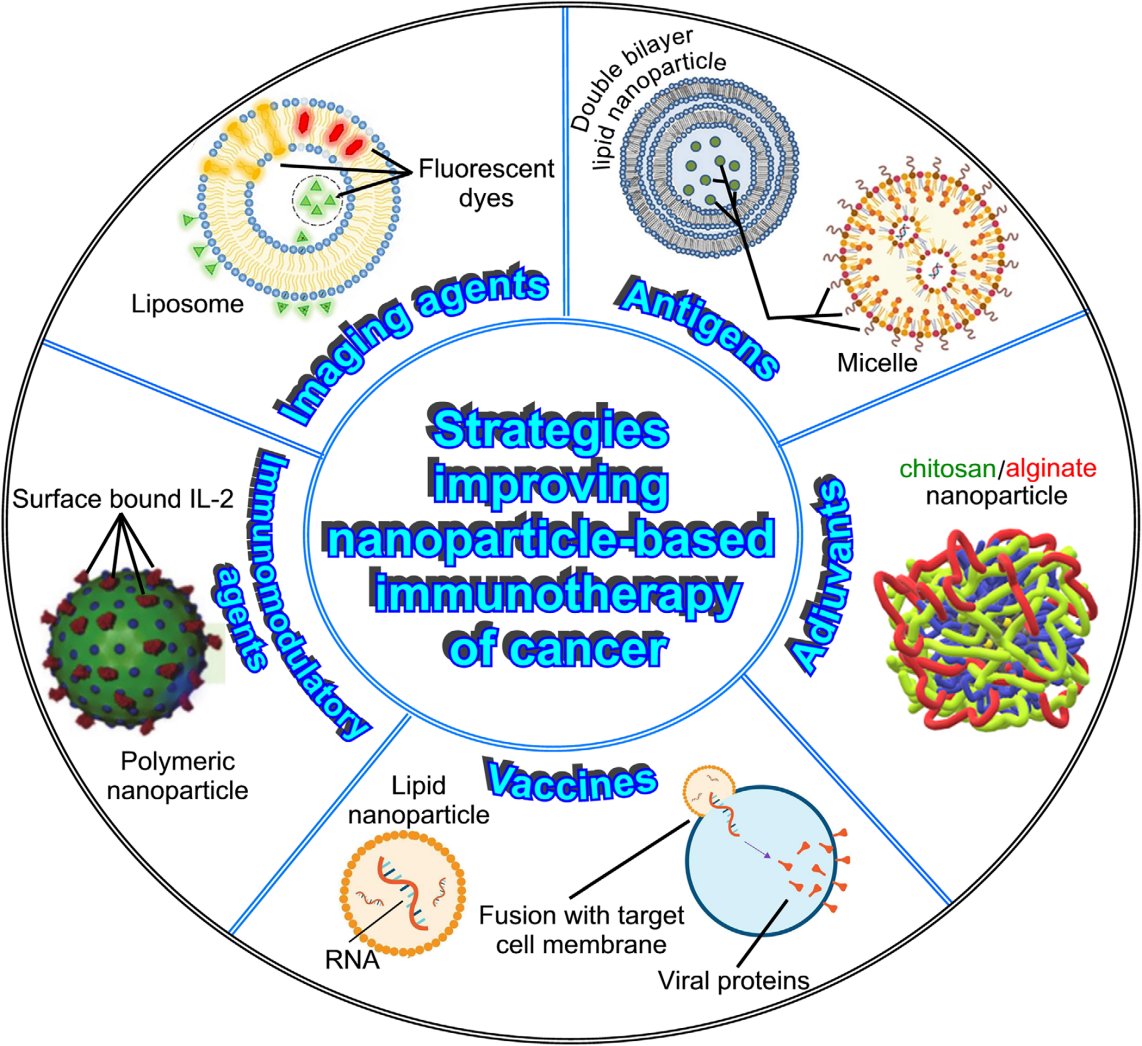
Graphic representation of the different strategies that can be used to achieve an effective antitumor immunity and improve therapeutic outcomes of nanoparticle‐based anticancer immunotherapy. (1) Antigen delivery. Double‐lipid bilayer nanoparticles can deliver tumor‐associated antigens to DCs, promoting the activation and expansion of cytotoxic T cells that can target cancer cells. (2) Adjuvant delivery. Nanoparticles made of polymers with immunostimulatory properties can boost antitumor immunity, and biopolymer‐based materials, such as polysaccharides (chitosan/alginate), are widely used due to their lack of allergic reactions and toxicity. (3) Vaccine platforms. Lipid nanoparticles can serve as vaccine platforms for cancer immunotherapy, since they can convey tumor antigens or DNA/RNA sequences encoding for tumor‐specific antigens, promoting an adaptive immune response against cancer cells. (4) Immunomodulatory agents. Polymer‐based nanoparticles can be loaded with immunomodulatory agents, such as cytokines (IL‐2) or CpG oligodeoxynucleotides, to promote antitumor immune responses. (5) Imaging and monitoring the effectiveness of immunotherapy. Nanoparticles, especially liposomes, loaded with specific materials and components that can respond to external and internal stimuli, such as enzymes or fluorochromes, can serve as imaging agents, allowing for monitoring immune responses and treatment efficacy. Created using Microsoft PhotoDraw (version 2.0.0.0822).

When using nanoparticles for anticancer immunotherapy, different strategies can be employed to enhance the immune response and improve therapeutic outcomes. Some of the most important are described below and represented in Figure 2.

### 3.1 Antigen delivery

Nanoparticles can deliver antigens to immune cells, promoting the activation of cytotoxic T cells that can target cancer cells. Nanoparticle‐based therapeutics, such as chemotherapy drugs or radio‐sensitizing agents that generate reactive oxygen species and oxidative stress when activated by radiation, can induce tumor cell death and in turn increase tumor neo‐antigen release.^77^

### 3.2 Adjuvant delivery

Immunostimulatory compounds can be incorporated into nanoparticles to boost antitumor immunity. NPs of biopolymer‐based materials, such as proteins (collagen, silk, albumin, gelatin, β‐casein, and zein), protein‐mimicking polypeptides, and polysaccharides (alginate, chitosan, starch, pullulan, and heparin), as well as commonly used polymers, including poly (lactic‐co‐glycolic acid) (PLGA), poly(glycolic acid) (PGA), and poly(lactic acid) (PLA), are widely used as cancer therapy vehicles due to their lack of allergic reactions and toxicity, as well as biodegradability.^78^ These biopolymers could induce innate immunity and stimulate potent T helper cell type 1 (Th1) immune responses to increase cytotoxic T lymphocytes (CTLs) activity.^78^ Among various biopolymers, poly (γ‐glutamic acid) γ‐PGA NPs can act as efficient antigen carriers, for antigen delivery to the DCs, and adjuvant system.^80^

### 3.3 Peptide and protein delivery

Nanoparticles can deliver peptides or proteins that act as ICIs and help in preventing cancer cells from evading immune detection and destruction. Peptides can be conjugated with nanoparticles such as liposomes to give them specific targeting ability, which can help them to be rapidly uptaken by specific cells.^81^ Compared with Abs, peptides are easier to synthesize and chemically modify, with better bioavailability, better biocompatibility, and lower immunogenicity, which make them a competitive option.^82^ Furthermore, peptides can self‐assemble and arrange into nanofibers, nanoparticles, or nanotubes due to their own aggregation ability or when modified by aggregation molecules. These aggregates can then be used as drug carriers with enhanced targeting and penetration.^83^

### 3.4 Vaccine platforms

Nanoparticles can serve as vaccine platforms for cancer immunotherapy. They can carry tumor antigens or DNA/RNA sequences encoding for tumor‐specific antigens, promoting an adaptive immune response against cancer cells.^84^ Nanoparticle‐based vaccines are being designed to raise T cell responses through antigen–adjuvant codelivery, multiantigen activation of DCs, and continuous antigen release. Other applications include in situ vaccination, which is designed to stimulate a strong local antitumor immunity involving both innate and adaptive immune cells, and as a result a durable systemic antitumor response, with artificial APCs or immune depots placed near tumors. By enhancing the dynamic interaction with lymphoid organ, through the optimization of the physicochemical properties (size, shape, charge, colloidal stability, and surface ligands), more nanovaccines have been successfully delivered directly to lymphoid organs, where their accumulation led to a rapid induction of antitumor immunity.^85^

### 3.5 Targeted delivery to immune cells

Surface modifications on nanoparticles enable targeted delivery to specific immune cells, such as macrophages, DCs, T cells, and B cells enhancing the precision of immunotherapeutic interventions. Nanoparticles can be engineered to reach and specifically deliver antigens to DCs and promote DC maturation and CTL activation.^86^ Conjugation with Abs or Ab fragments, that recognize specific receptors on immune cells, is one of the most common surface modifications of NPs. Anti‐CD11c Abs are often used to target DCs, as CD11c is predominantly expressed by this cell type. Similarly, anti‐CD4 Abs are used for targeting CD4^+^ T cells,^87^ whereas anti‐CD206 Abs can selectively target macrophages,^88^ especially M2 macrophages, which are involved in tumor promotion. Nanoparticle conjugation with peptide ligands can be used to target receptors expressed on immune cells, as in the case of the arginylglycylaspartic acid (RGD) peptide, which binds to β2 integrins, such as α_M_β_2_ (i.e., Mac‐1, CD11b/CD18) and α_L_β_2_, (i.e., lymphocyte function‐associated antigen 1, CD11a/CD18) expressed on macrophages and DCs.^89^, ^90^ Nanoparticle conjugation with carbohydrate ligands, such as mannose, can be used for targeting immune cells expressing carbohydrate‐binding receptors, known as lectins. Mannose‐functionalized nanoparticles, which target mannose receptors (CD206) on DCs and macrophages,^91^ are useful to enhance vaccine responses, or to deliver antigens to APCs. Nanoparticle conjugation with aptamers, which consist in short, single‐stranded nucleic acids that fold into specific three‐dimensional structures, allows binding to target molecules with high specificity.^92^ Aptamers that bind to surface proteins such as PD‐L1 (programmed death‐ligand 1)^93^ or PD‐1 (programmed death‐1),^94^ can be conjugated to nanoparticles for targeted delivery to cancer or immune cells, such as T lymphocytes, B, and natural killer (NK) cells involved in cancer immunotherapy. Nanoparticles can be functionalized with ligands that bind to Fc receptors, which are expressed on different immune cells, including macrophages, DCs, and B cells. Nanoparticles coated with Fc fragments can target Fcγ receptors (FcγRs) on immune cells, promoting phagocytosis by macrophages or activation of DCs, which is desirable in cancer immunotherapy or vaccine delivery.^95^, ^96^

### 3.6 Combination therapies

Nanoparticles facilitate the delivery of multiple therapeutic agents simultaneously, allowing for combination therapies. This approach can enhance the synergistic effects of immunotherapy, such as ICIs, that is, Abs, RNAs, peptides, or small molecules, which can block immune checkpoint proteins, and other treatment modalities, such as chemotherapy.^97^

### 3.7 Immunomodulatory agents

Nanoparticles can carry immunomodulatory agents to the tumors and TME to promote antitumor immune responses. Immunostimulatory agents include cytokines, such as IL‐2, which has been approved by the US FDA for treating metastatic melanoma and renal cell carcinoma^98^, ^99^ or IFNs, such as IFNα, which promotes major histocompatibility complex class I expression leading to better tumor antigen recognition, and a Th1 shift in host immunity, enhancing cell‐mediated cytotoxicity, and has been approved by the US FDA as an adjuvant therapy for stage III melanoma.^100^ Other immunostimulatory agents are represented by CpG oligodeoxynucleotides, some of which can be used as potent Th1‐biasing adjuvants and have demonstrated great potential in cancer therapy.^101^

### 3.8 Long‐lasting immune activation

Controlled release of immunotherapeutic agents from nanoparticles can result in a sustained and prolonged immune response, improving the durability of the anticancer effects. The blood circulation time of nanomaterials can be increased through surface coating, for example, the coating of liposomes with hydrophilic polymers, such as PEG70, with an optimal PEG density of approximately 10 mol%. Nanomaterials inherently interact with phagocytic myeloid cells and are thus ideal platforms with which to regulate trained immunity, that is, long‐term functional reprogramming of the innate immune cells, which confer protection against a second homologous/heterologous challenge.^102^, ^103^

### 3.9 Minimizing immune evasion

Nanoparticles’ surface can be coated with synthetic polymers, such as PEG that may enable evasion from phagocytic clearance, which is essential for their long‐term circulation and biodistribution.^104^ Nanoparticles can also be designed to target or polarize macrophages from M2 tumor growth‐promoting phenotype toward M1 cancer cell‐killing phenotype.^105^ The physical properties of nanomaterials, such as size, structure, shape, charge, mechanical strength, and hydrophobicity, can directly or indirectly influence immune cell functions and modulate immune responses.^106^ In addition, nanoparticles can be designed to counteract mechanisms by which cancer cells evade the immune system either (1) by targeting immunosuppressive cells or molecular pathways or (2) inducing ICD through the activation of CTLs via APCs, thus reshaping the immunosuppressive TME.^107^

### 3.10 Imaging and monitoring the effectiveness of immunotherapy

Some recently developed nanoparticles, such as smart nanoparticles, including polymeric nanoparticles, dendrimers, micelles, liposomes, protein nanoparticles, cell membrane nanoparticles, GNPs, iron oxide nanoparticles, QDs, CNTs, based on specific materials and components that can respond to external and internal stimuli (such as enzyme, pH, temperature, as well as optical and magnetic regulation, etc.) can serve as imaging agents, allowing for monitoring in real‐time immune responses and treatment efficacy, therefore providing patients with individualized treatment options.^108^

Challenges in the field include optimizing nanoparticle properties, addressing potential toxicity, and understanding the complex interactions within the TME. Nevertheless, ongoing research aims to overcome these challenges and to unlock the full potential of nanoparticles in advancing anticancer immunotherapy.

## 4 NANO‐BASED CRISPR/CAS9 DELIVERY FOR MOLECULAR TARGETED THERAPY OF CANCER

**FIGURE 3:**
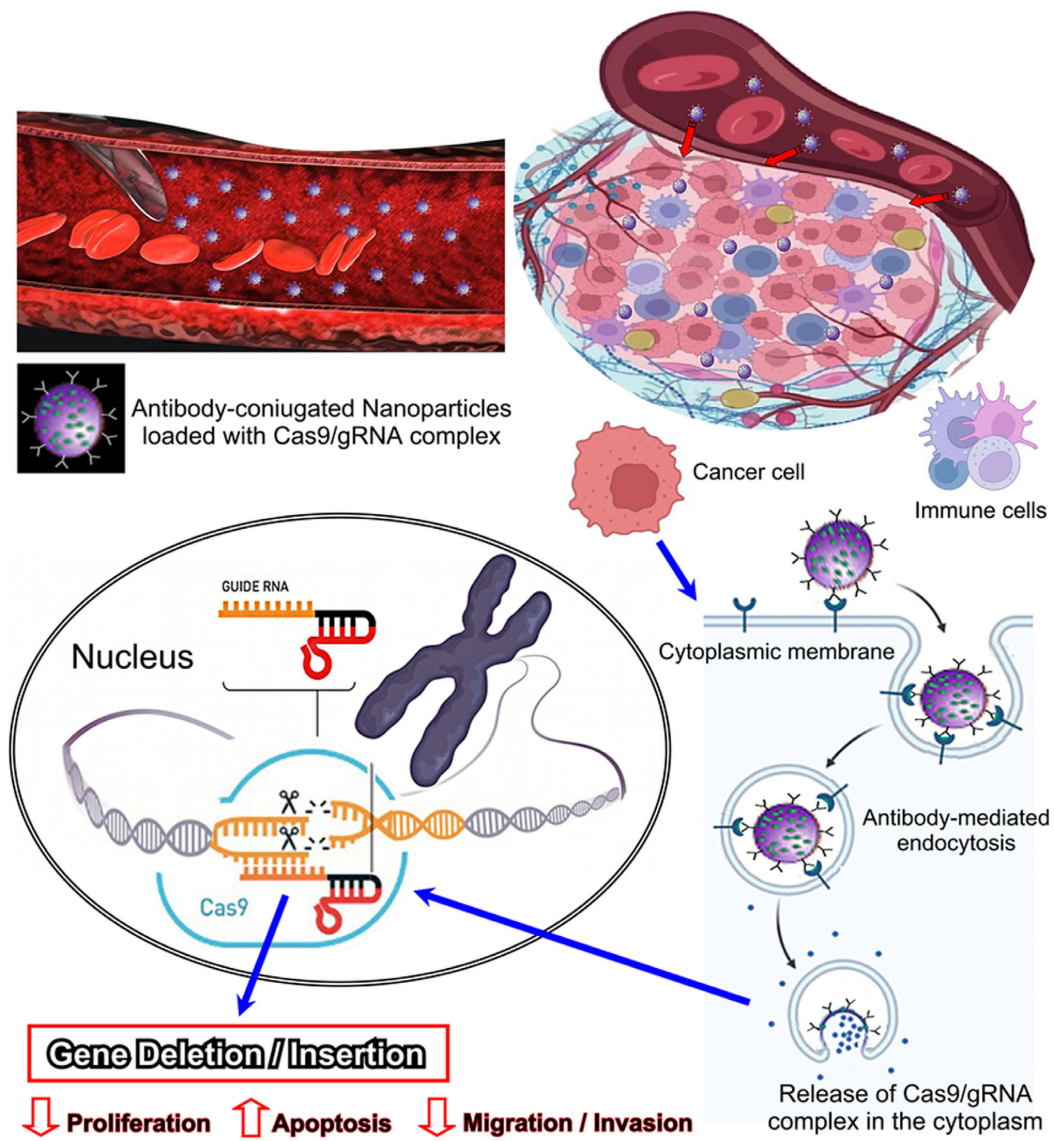
Graphic representation summarizing the key points of nano‐based CRISPR/Cas9 delivery for molecular targeted therapy of cancer. Created using Microsoft PhotoDraw (version 2.0.0.0822).

The recent development of the clustered regularly interspaced short palindromic repeats/CRISPR‐associated nuclease (CRISPR/Cas9) technology represents a real breakthrough in genetic engineering, which has great application potential for the treatment of different diseases.^109^, ^110^ Combining nanoscience with CRISPR/Cas9 genome engineering, which is based on Cas9/single (s) guide (g) RNA ribonucleoprotein complexes (RNPs) for transcriptional manipulation and gene editing as well as for epigenetic modulation, holds great promise for the development of targeted anticancer therapies.^111^ This innovative and versatile approach that leverages the precision of CRISPR/Cas9 gene editing, which allows for the precise modification of specific genes within the genome, along with the unique properties of nanomaterials to deliver therapeutic payloads specifically to cancer cells, can enhance targeted delivery, reduce off‐target effects, and improve overall therapeutic efficacy, while minimizing damage to healthy tissues. Specific genetic mutations of tumor suppressor genes, and activated oncogenes driving cancer progression or therapy resistance, can be repaired, or deleted, respectively, by using gRNA sequences for CRISPR‐mediated gene knockouts or knock‐ins. The CRISPR/Cas9 system can be loaded onto or integrated within nanoparticles, such as liposomes,^112^ polymeric nanoparticles, or other nanocarriers that protect the CRISPR payload from degradation and facilitate its delivery to the target cells^113^, ^114^, ^115^ (Figure 3).

Nanoparticles can be designed to selectively accumulate in tumor tissues, due to the enhanced permeability and retention (EPR) effect, which is a characteristic of the leaky blood vessels found in tumors. Alternatively, functionalization of nanoparticles, by ligand or Ab conjugation, can enhance tumor targeting and favor their uptake and internalization in cancer cells.

Here's an overview of how precision anticancer nanotherapy using CRISPR/Cas9 technology might work.

### 4.1 Identification of target genes

Activation/overexpression of specific oncogenes, associated with cancer development and progression, such as *KRAS, SMAD4* and *EGFR*,^116^ chemotherapy resistance genes, such as *NRF2*,^117^ *ABCB1*, and *MDR1*,^118^ metabolism‐associated genes, such as *hypoxia inducible factor 1α* and *GLUT‐1*,^119^, ^120^ and stemness‐related genes, such as *CD133*, *CD44*, *NANOG*, *CXCR4*, *CECAM5*, and *WNT*,^118^ or silencing/deficiency/mutation of tumor suppressor genes, such as *NF2*,^121^ *PTEN*,^122^ and *adenomatous poliposis coli gene*,^123^ can be targeted by the CRISPR/Cas9 gene editing system. Interestingly, immune checkpoint gene, such as *PD‐1*, can be knocked out by CRISPR/Cas9 genome editing in peripheral T lymphocytes, which are then expanded ex vivo and then transfused back into the patient. Five clinical trials testing *PD‐1* knockout for the treatment of metastatic non‐small cell lung cancer, prostate, bladder, esophageal, and renal cell cancers have been registered.^124^ Furthermore, CRISPR/Cas9‐based gene editing may overcome many of the limitations related to the use of chimeric antigen receptor (CAR) T‐cells (which consists in synthetic receptors comprised of antigen recognition, signaling, and costimulatory domains used to reprogram T‐cells to target and destroy tumor cells), such as T‐cell exhaustion, lack of CAR T‐cell persistence and cytokine‐related toxicities, by targeting negative regulators of T‐cell function, directing therapeutic transgenes to specific genomic loci, and generating reproducibly safe and potent allogeneic universal CAR T‐cell products for on‐demand cancer immunotherapy.^125^

Targeting of tumor promoting cytokine genes, using nanodelivery systems is a further area of active research aimed at boosting immunotherapy and chemotherapy.^99^, ^126^ We recently demonstrated that CRISPR/Cas9‐mediated targeting of the interleukin (IL)‐30 gene, which promotes an immunosuppressive TME and regulates self‐renewal and/or proliferation, migration, and gene expression profiles in prostate and colorectal cancer stem and differentiated cells, substantially inhibits tumor progression and prolongs survival in preclinical xenograft models, substantiating data on the efficacy of the antitumor and immunoregulatory potential of the nano‐based CRISPR genome editing approach.^127^, ^128^

### 4.2 Designing CRISPR/Cas9 constructs

Customized CRISPR/Cas9 constructs are designed to specifically target and modify the identified cancer‐related genes. After selecting the gene(s) to be targeted, based on the tumor type and genetic profiling of the specific cancer, critical steps for designing such constructs involve (a) designing the gRNA, (b) choosing the Cas9 variant, and (c) choosing the delivery system.^129^

- Designing gRNAs that direct the Cas9 nuclease to specific sequences within the gene must comply with the specificity requirement. The gRNA should bind specifically to the target gene sequence (a region critical for the function of the gene, e.g., catalytic domains, mutations) to avoid off‐target effects. Off‐target activity can be minimized by choosing sgRNAs with minimal sequence similarity to other genes and by using predictive tools to assess potential off‐target sites.^130^ Importantly, it must bind to a target site adjacent to a protospacer adjacent motif sequence (usually “NGG” for Streptococcus pyogenes Cas9) and should efficiently guide the Cas9 to cleave the target DNA.^131^ Several bioinformatics tools, such as Benchling, CRISPOR, or CHOPCHOP, can be used to design and optimize gRNA sequences with minimal off‐target effects.^132^
- The choice of the Cas9 variant depends on the therapeutic goal. Wild‐type Cas9 cuts both DNA strands and leads to knockouts by nonhomologous end joining (NHEJ)^133^; Nickase Cas9 (Cas9n) introduces single‐strand breaks, reducing off‐target effects when combined with two gRNAs; dead Cas9 is used for transcriptional regulation (CRISPR interference and CRISPR activation) of the gene without cutting DNA; base editors, obtained from the fusion of Cas9 with deaminase enzymes, are used for precise base editing without introducing double‐strand breaks (DSBs). For antitumor therapy, wild‐type Cas9 is typically used to knockout oncogenes, while base editors might be employed for point mutations. For oncogene knockout, the goal is typically to introduce frameshift mutations that lead to loss of function. Cas9‐induced DSBs followed by NHEJ will cause small insertions or deletions (indels), disrupting the target gene. For tumor suppressor gene correction, homology‐directed repair^133^ or base editing can be used to correct inactivating mutations. This approach requires codelivery of a repair template with homology arms flanking the mutation site.^134^
- The CRISPR/Cas9 system is typically delivered via plasmids or viral vectors such as adeno‐associated virus (AAV), lentivirus, or adenovirus. When designing these constructs, it must be considered that strong and tumor‐specific promoters (e.g., hTERT, survivin) can drive the expression of Cas9 and gRNA specifically in cancer cells. AAV and lentiviruses have good transduction efficiency, but their payload sizes are limited, so construct size must be optimized. Finally, targeting multiple genes simultaneously is advantageous for certain tumors, so the construct may include multiple gRNAs.^135^, ^136^

### 4.3 Encapsulation into nanoparticles

Encapsulating CRISPR components into nanoparticles allows for enhanced delivery and efficacy of gene‐editing therapies.^137^ This strategy entails addressing many challenges associated with the delivery of CRISPR–Cas9 systems, such as degradation by nucleases, immune recognition, off‐target effects, and inefficient delivery to target cells or tissues.

Different types of nanoparticles can be used to encapsulate and deliver CRISPR components: (a) lipid‐based nanoparticles (LNPs) are often used in delivering messenger RNA (mRNA) and other nucleic acids, as they can encapsulate Cas9 mRNA, gRNA, or the whole RNP complex.^138^ LNPs protect the CRISPR components from degradation and facilitate cell membrane fusion and endosomal escape^139^; (b) polymeric nanoparticles made of polymers like polyethyleneimine (PEI), PLA, and PLGA,^140^ provide a tunable release profile and can enhance the stability and solubility of the CRISPR–Cas9 system. The main advantage of PEI lies in its efficient endosomal escape.^114^ Moreover, the difficulty of PEI biodegradation and its cytotoxicity have limited its widespread application as a nucleic acid delivery material^141^; (c) inorganic nanoparticles, which include gold nanoparticles (GNPs), silica nanoparticles, or magnetic nanoparticles,^112^ can also provide imaging capabilities, magnetic guidance, or controlled release triggered by external stimuli (e.g., light or magnetic fields); (d) viral vectors, such as lentivirus, adenovirus, and AAV, are commonly used for CRISPR delivery.^142^ They can be engineered to efficiently deliver CRISPR components into specific cell type, although concerns about immunogenicity and integration into the host genome have shifted interest toward nonviral nanoparticle systems.^143^

Different strategies can be used to encapsulate CRISPR components into nanoparticles, depending on the type of nanoparticle and the nature of the CRISPR system^144^, ^145^: (a) physical encapsulation of the CRISPR payload inside LNPs, which are widely used for their efficiency, safety, and versatility, is carried out by using microfluidic mixing or solvent evaporation techniques.^146^ The negatively charged DNA or mRNA is encapsulated in the aqueous core of the liposome. In the case of preformed Cas9 protein and gRNA complexes, they can be encapsulated in the liposome core or adsorbed to the surface of cationic liposomes^147^; (b) surface functionalization, which is used when Cas9 or gRNA can be adsorbed (through electrostatic interactions, van der Waals forces, or hydrophobic interactions) or conjugated (using crosslinkers that react with functional groups, e.g., amines, carboxyls, on the Cas9 protein or gRNA) onto the surface of nanoparticles, usually inorganic nanoparticles like gold, where surface chemistry can be tailored for efficient conjugation. Although, the chemical modification of Cas9 or gRNA, may compromise their functionality; (c) layer‐by‐layer assembly, which involves alternating layers of charged polymers and CRISPR components, building up a nanoparticle through electrostatic interactions. It allows for precise control over the loading and release of CRISPR agents.^148^, ^149^

### 4.4 Targeted delivery to cancer cells

Targeted delivery of nanoparticles to cancer cells aims to enhance the specificity and efficacy of treatments, minimize off‐target effects, and reduce toxicity to healthy tissues. Two main approaches can be used for cancer cell targeting: (a) passive targeting through the EPR effect due to the leaky vasculature and impaired lymphatic drainage of the tumor, which enables nanoparticles to accumulate preferentially in the tumor site.^150^ Although the EPR effect is widely used, it may not be uniformly effective across all tumor types due to variations in vascularization, and it lacks precision in targeting specific cancer cells; (b) active targeting by functionalizing nanoparticles with ligands that bind to receptors overexpressed on cancer cells.^150^ Strategies for active targeting include: (1) ligand–receptor binding. Nanoparticles can be coated with ligands such as Abs, peptides, aptamers, or small molecules that recognize specific receptors on cancer cells; (2) monoclonal Abs (e.g., anti‐HER2 Abs for breast cancer and anti‐PSCA Abs for prostate cancer) which can be attached to nanoparticles to target overexpressed antigens on cancer cells; (3) short peptides like RGD, which can be used for targeting tumors with high expression of integrins^151^ such as αvβ3, α5β1, α6β4, etc…^152^, ^153^; (4) aptamers, which can bind specifically to cancer cell surface proteins, with high affinity and specificity^92^; (5) folate, which is commonly used to target folate receptor‐positive cancer cells, such as ovarian and breast cancers^154^, ^155^; (6) pH‐sensitive nanoparticles, which contain ionizable polymers [poly(l‐histidine or poly(acrylic acid)], or pH‐labile linkers (hydrazones or imine bonds), designed to release their payload (undergoing structural changes, such as swelling, disassembly, or collapse), or enhance cell binding or uptake (through charge changes or exposure of targeting moieties), in response to the pH of the TME, which is typically lower (around 6.5–6.8, due to hypoxia and increased metabolic activity) compared with the physiological pH (∼7.4) of healthy tissues^156^, ^157^, ^158^; (7) magnetic and light guided nanoparticles (e.g., iron oxide nanoparticles), which can be directed to tumors using external magnetic fields.^159^, ^160^ Similarly, photothermal or photodynamic therapy can guide nanoparticles to tumors using light‐based activation.^161^, ^162^, ^163^

### 4.5 Release and gene editing

Once the nanoparticles reach the cancer cells, they release the CRISPR/Cas9 constructs. The Cas9 enzyme then cuts the target DNA sequence, and the cell's natural repair mechanisms either introduce mutations (gene knockout) or facilitate the insertion of the corrected genetic material. Targeted enzymatic digestion mediated by the CRISPR machinery can be harnessed as a diagnostic tool to identify cancer‐specific sequence changes. Microsatellites, a diagnostic marker in cancers,^164^ can be sensitively detected using CRISPR‐mediated digestion targeting the short tandem repeats, which make up microsatellites. When paired with duplex sequencing that incorporates double‐stranded DNA barcodes to prevent errors in sequencing, Cas9‐mediated fragmentation allows for targeted sequencing of genomic regions even with very little DNA input (termed CRISPR‐duplex sequencing, DS).^165^ CRISPR‐DS is currently being evaluated in a clinical trial for the detection of p53 mutations in ovarian tumors.^166^

The use of nanotherapy, to convey CRISPR/Cas9 for genome editing specifically in tumor cells, may lead to a big step forward in precision oncology^167^; however, before its translation into the clinic, there are still challenges to overcome, such as optimizing delivery efficiency, addressing potential immunogenicity, reducing off‐target effects, ensuring the long‐term safety of the therapy, and navigating regulatory and ethical considerations. While clinical trials testing the safety and efficacy of anticancer therapeutics based on the CRISPR/Cas9 system are ongoing, trials testing CRISPR/Cas9 loaded nano‐based formulations are still awaited.^168^

## 5 DEVELOPMENT OF PATIENT'S TUMOR TAILORED NANOTHERAPY USING ORGAN‐ON‐CHIP TECHNOLOGY

Assessing anticancer nanotherapy for precision oncology using organ‐on‐a‐chip (OOC) technology involves evaluating the effectiveness, specificity, and safety of the nanotherapeutic approach within a microscale model that mimics the physiological conditions of organs and tissues. OOC platforms are microfluidic devices that simulate the physiological and mechanical properties of human organs, providing a more accurate representation of in vivo conditions compared with traditional cell culture methods.^169^ This technology can help assess precision, targeting, and efficacy of nanodrugs in cancer treatment at the preclinical level.^170^ Key aspects of the development of anticancer nanotherapy using OOC are discussed below.

### 5.1 Integration of patient‐specific data into the OOC model to improve the precision of the nanotherapy in a personalized medicine approach

Incorporating in the OOC models, 3D multicellular hetero‐spheroids, which include patient‐derived cancer, stromal, and immune cells into an extracellular matrix‐like collagen gel, allows the development of personalized treatment strategies by evaluating the response of individual tumors to different anticancer nanotherapies.^171^

### 5.2 Microenvironment replication

Verification of the OOC technology's capability to replicate the key features of the TME, such as cellular interactions, extracellular matrix composition and stiffness, interstitial fluid flow and gradients of oxygen (hypoxia), nutrients, and waste products. Examination of how well the OOC model mimics the physiological conditions of the targeted tumor to ensure relevance to the in vivo scenario.^172^

### 5.3 Microfluidic drug delivery

Integrated microfluidic channels enable precise control of fluid flow, nanoparticle and drug dosage, spatial‐temporal dynamics of nanoparticle exposure to the complex TME, and metastatic sites played within the OOC platform. Nanoparticle transport, distribution, and impact on the TME can be investigated in a context that mimics blood flow, lymphatic drainage, nutrient transport, waste removal, and other patho‐physiological aspects of living neoplastic tissues^169^, ^173^ and metastatic sites.^174^

### 5.4 Real‐time monitoring of nanoparticle targeting and therapeutic efficacy

Sensors or imaging techniques can be incorporated into the OOC system for real‐time monitoring of therapeutic effects, cellular responses, and nanoparticle behavior.^175^, ^176^ Advanced microscopy or imaging modalities can be used to visualize the interactions between nanoparticles and cancer cells and to evaluate the ability of the anticancer nanotherapy to target specific cancer cells and assess the delivery efficiency, including nanoparticle transport, cellular uptake, and release of therapeutic agents.^175^, ^176^ Nanotherapy's ability to induce apoptosis or inhibit proliferation or invasion and migration of cancer cells within the OOC model can be analyzed by flow cytometry and laser scanning confocal (LSC) microscopy.^175^, ^176^

Nanotherapy's impact on the size, phenotype, and genotype of the tumor, reproduced with 3D organoids or spheroids,^177^, ^178^ can be assessed by LSC, flow cytometric analyses, and real‐time RT‐PCR or single cell sequencing.^179^ High‐content screening measuring biological parameters and imaging platforms that are designed for single‐cell or whole organoids analysis and lightning‐fast time‐to‐data, perform unbiased spontaneous phenotyping with live cells from the patient's tumor‐derived organoids.^180^

### 5.5 Biodistribution and pharmacokinetics

OOC technology allows to analyze the biodistribution of nanoparticles within the OOC compartments, tracking their movement and accumulation. The sustained and controlled release over time of anticancer nanotherapies is ensured by a pulsatile and dynamic flow, mimicking systemic circulation, that allows to evaluate pharmacokinetic parameters to understand how the nanotherapy behaves over time in the simulated TME.^181^, ^182^

### 5.6 Off‐target effect and safety assessment

OOC models allow the investigation of potential off‐target effects on healthy cells within the OOC, ensuring that the nanotherapy is selective for cancer cells. They permit the evaluation of the safety and potential toxicity of anticancer nanotherapies, and assessment of their impact on healthy tissues.

### 5.7 Use of platforms for high‐throughput drug screening and machine learning approaches

To identify the most effective anticancer nanotherapies and to optimize nanoparticle formulations and treatment protocols according to the results obtained from the OOC models.^183^ Effects on the TME or normal tissues, of combining different anticancer agents, such as chemotherapy drugs, immunotherapeutics, and targeted nanoparticles, can be monitored providing relevant information for precision medicine.

### 5.8 Reproducibility and validation

OOC technology allows to conduct experiments with replicates, under monitored conditions that ensure the reproducibility of the results, and to validate findings by comparing them with established in vitro and in vivo data.

By combining the precision of nanotherapy with the physiological human relevant OOC models, researchers can bridge the gap between traditional in vitro studies and more complex in vivo experiments, providing a more realistic and efficient platform for assessing the safety and efficacy of anticancer nanoformulations. Given the rapid advances of the OOC field, the list of relevant endpoints to be extracted from OOCs needs to be constantly updated to integrate new, promising tissue‐level read‐outs with high clinical relevance.^184^

## 6 APPROVED NANO‐BASED ANTITUMOR PHARMACEUTICALS

Anticancer nanopharmaceutics is a rapidly evolving field.^185^ The majority of nanopharmaceuticals for cancer treatment approved by the US FDA and the European Medicines Agency (EMA) are lipid‐based and protein‐based DDS. Innovations in liposome technology, and the incorporation of micelles, polymeric nanomaterials, and inorganic‐based nanoparticles, together with the application of targeting with a variety of ligands, have provided a new generation of nanopharmaceuticals currently in testing in clinical trials, alone or in combination with conventional treatments.^186^, ^187^ Notable examples of nanopharmaceuticals that are being explored are the following.

### 6.1 Liposomal chemotherapy

Liposomes are nanoscale phospholipid bilayered vesicles endowed with an aqueous core that can encapsulate hydrophilic chemotherapeutic agents, improving their pharmacokinetics and biodistribution, and reducing side effects. Examples include Doxil (liposomal DOX) and Onivyde (liposomal irinotecan).^188^, ^189^

### 6.2 Nanoparticle albumin‐bound (nab) technology

The core idea of nab technology is to bind therapeutic agents to albumin nanoparticles (usually ∼100–150 nm in size) to improve the solubility, bioavailability, and tumor targeting of the drugs. Abraxane, a nanoparticle albumin‐bound (nab) formulation of paclitaxel, improves drug solubility and delivery. Albumin‐based nanocarriers could potentially overcome cancer drug resistance through bypassing drug efflux, enhancing drug uptake, and improving tumor accumulation. Moreover, albumin nanocarriers improve the stability of various therapeutic cargos, for instance, nucleic acids, and allow their systemic administration.^190^

### 6.3 Polymeric nanoparticles

Biodegradable and biocompatible polymers, such as the US FDA‐approved PLGA, are used to synthesize polymeric nanoparticles, which can be designed to encapsulate and deliver various anticancer drugs. They offer controlled release and targeted drug delivery. Examples include Genexol‐PM (paclitaxel‐loaded polymeric nanoparticles), Doxil (DOX‐loaded polymeric nanoparticles), and Nanoxel (docetaxel‐loaded polymeric nanoparticles).^188^, ^189^, ^191^, ^192^

### 6.4 Silica nanoparticles

There are three main types of silica nanoparticles: solid, nonporous, and mesoporous.^193^ To date, the MSNPs are considered the most promising for future clinical use.^193^, ^194^ They are composed of a honeycomb‐like porous structure, consisting of hundreds of empty channels that range in diameter from 2 to 50 nm.^193^ The fabrication of MSNPs is simple and cost efficient and their size can be easily tuned, thus permitting endocytosis with negligible cytotoxicity.^193^ They are highly resistant to pH, mechanical stress, heat, and hydrolysis‐induced degradations, and, in addition to a large surface area (>800 m^2^/g), MSNPs also possess high pore volume (>0.9 cm^3^/g), thus allowing high loadings of drug molecules.^193^, ^194^ These characteristics make them the material of choice for targeted delivery of active pharmaceuticals for cancer therapy and other treatments, as evidenced by the rising number of scientific articles involving their use as drug carriers.^193^, ^194^, ^195^, ^196^

### 6.5 Dendrimer‐based therapies

Dendrimers are highly branched nanoparticles that can be engineered to carry drugs. They offer a high degree of control over drug release kinetics. Due to their multivalent structure, dendrimers can carry many drug molecules, enhancing the therapeutic payload delivered to the tumor. Dendrimer‐based formulations of anticancer drugs, such as dendrimer–DOX conjugates and dendrimer–methotrexate conjugates, are being explored.^197^

### 6.6 Gold nanoparticles

GNPs can be used for drug delivery and imaging purposes, due to their impressive optical properties, and have been a forerunner in bioengineered cancer therapy. They have tunable properties and can be functionalized for targeted drug delivery. The photothermal property of nanoparticles, especially of gold nanorods, causes absorption of the light incident by the light source, and transforms it into heat, resulting in tumor cell destruction. Applications of this theranostic system include molecular detection, biological imaging, and cancer cell targeting.^198^

### 6.7 RNA nanoparticles

RNAs, such as siRNA or mRNA, are being investigated for their potential in gene therapy and in silencing cancer‐related genes. Currently, there are 11 marketed products based on antisense oligonucleotides, aptamers, siRNAs, and many others are in the pipeline. RNA‐based gene therapy requires therapeutic RNA to function inside target cells without eliciting unwanted immune responses. Encapsulation into conjugated polymer‐based or lipid‐based nanocarriers^199^ may overcome this issue and insure its targeted delivery.^200^

### 6.8 Carbon nanotubes

CNTs have unique properties that make them suitable for drug delivery. Due to their excellent optical property, thermal and electronic conductivity, easy functionalization ability, and high drug loading capacity, CNTs can be applied in a multifunctional way for both cancer treatment and diagnosis. Their targetability to intracellular and extracellular components of the TME makes CNTs one of the most promising tools for cancer theranostics.^53^

### 6.9 Immunoliposomes and immunonanoparticles

Liposomes (spherical vesicles composed of lipid bilayers) or nanoparticles (much smaller than liposomes and made of gold, silica, or polymers) functionalized with Abs (such as, anti‐EGFR, ‐HER2, ‐PSCA, ‐PSMA) or Ab fragments, designed to selectively target the antigen expressed on cancer cells,^201^ can be used for tumor‐specific drug delivery. Their binding to cancer cells favors tumor uptake and accumulation, while reducing off‐target effects and systemic toxicity.^201^ A range of immunoliposome‐based drugs are currently available for the treatment of a variety of diseases, including breast and ovarian cancer, Kaposís sarcoma, and acute lymphoblastic leukemia.^202^

### 6.10 Magnetic nanoparticles

Magnetic nanoparticles can be used for targeted drug delivery and hyperthermia (heating) treatments. They can be guided to specific tumor sites using external magnetic fields. The latest clinical trial testing the magneto‐thermal therapy, named NanoTherm® therapy, is the world's first magnetic iron oxide nanoparticle (MION)‐based therapy, designed for the treatment of prostate cancer (NCT02033447) and glioblastoma (DRKS00005476).^203^, ^204^

### 6.11 Cancer nanovaccines

Nanoparticle‐based cancer vaccines, often utilizing lipid nanoparticles, are being developed to stimulate the immune system to recognize and attack cancer cells.^205^, ^206^ Nanovaccines containing tumor antigens have great bioavailability and pharmacokinetic qualities, which are crucial for inducing a potent and durable anticancer immune response. They enable cross presentation enhancement and intracellular antigen delivery modulation. Antigens adsorbed on cationic dendrimer NPs are more efficiently delivered to DCs while activating them and causing them to secrete cytokines, such as IL‐1β and IL‐12. DCs are essential for the coordination of the innate and adaptive immune systems through the absorption, processing, and presentation of epitopes to naive T cells. Lipid, polymeric, and inorganic NPs can effectively stimulate the growth of CD8^+^T cells by antigen cross‐presentation.^207^

Nanopharmaceuticals currently on the market are categorized into crystalline, liposome‐derived, polymeric‐type, protein‐based, and metal‐based.^208^, ^209^ Nanopharmaceuticals approved and marketed for cancer therapy are listed in Table 1; those currently undergoing clinical trials are listed in Table 2.

**TABLE 1:** Nanomedicine drugs approved for cancer treatment.^209^

Product name	Drug	Technology	Cancer type	Approval
*Abraxane*	Paclitaxel	Protein carrier	Various cancers including pancreatic cancers	2005 (US FDA)
*Ameluz*	5‐Aminolevulinic acid	Gel containing 5‐aminolevulinic acid, E211, SoyPC, and PG	Superficial and/or nodular basal cell carcinoma	2011 (EMA)
*Apealea*	Paclitaxel	Polymeric micelles	Ovarian, peritoneal and fallopian tube cancer	2018 (EMA)
*DaunoXome*	Daunorubicin	Liposome	AIDS‐related Kaposi's sarcoma	1996 (US FDA)
*Depocyt*	Cytarabine	Liposome	Lymphomatous malignant meningitis	1999 (US FDA)
*Doxil (Caelyx)*	Doxorubicin hydrochloride	Pegylated liposome	Ovarian cancer and AIDS‐related Kaposi's sarcoma	1995 (US FDA)
*DPH107*	Paclitaxel	Lipid nanoparticles	Advanced gastric cancer	2016 (South Korea)
*Eligard*	Leuprolide acetate	Polymeric nanoparticles	Advanced prostate cancer	2002 (US FDA)
*Genexol‐PM*	Paclitaxel	Polymeric micelle	Non‐small cell lung cancer	2006 (South Korea)
*Kadcyla*	DM1	Trastuzumab, covalently linked to DM1 via the stable thioether linker MCC	HER2^+^ breast cancer	2013 (US FDA, EMA)
*Lipo‐Dox*	Doxorubicin	Liposome	Kaposi's sarcoma, breast and ovarian cancer	1998 (Taiwan)
*Lipusu*	Paclitaxel	Liposome	Breast cancer, non‐small cell lung cancer	2013 (EMA)
*Marqibo*	Vincristine	Liposome	Leukemia	2012 (US FDA)
*Mepact*	Muramyl tripeptide phosphatidyl ethanolamine	Liposome	Nonmetastatic osteosarcoma	2009 (EMA)
*Myocet*	Doxorubicin	Liposome	Breast cancer	2000 European Medicines Agency (EMA)
*NanoTherm*	Fe_2_O_3_	Nanoparticles of superparamagnetic iron oxide coated with amino silane	Glioblastoma, prostate, and pancreatic cancers	2013 (EMA)
*Nanoxel*	Docetaxel	Polymeric micelle	Breast and ovarian cancers, non‐small cell lung cancer, and AIDS‐related Kaposi's sarcoma	2006 (India)
*NBTXR3 (Hensify)*	Hafnium oxide nanoparticles stimulated with external radiation	Hafnium oxide nanoparticles	Locally advanced squamous cell carcinoma	2019 (EMA)
*Oncaspar*	l‐Asparaginase	PEGylated conjugate	Acute lymphoblastic leukemia	2006 (US FDA)
*Onivyde*	Irinotecan	Liposome	Pancreatic cancer	2015 (US FDA)
*Ontak*	Denileukin diftitox	Recombinant DNA derived cytotoxic protein	Cutaneous T‐cell lymphoma	1999 (US FDA)
*Pazenir*	Paclitaxel	Paclitaxel formulated as albumin bound nanoparticles. Powder for dispersion and infusion	Metastatic breast cancer, metastatic pancreatic cancer, non‐small cell lung cancer	2019 (EMA)
*Vyxeos*	Daunorubicin and cytarabine	Liposome	Acute myeloid leukemia	2017 (EMA)
*Zinostatin stimalamer*	Styrene maleic anhydride neocarzinostatin	Polymer protein conjugate	Primary unresectable hepatocellular carcinoma	1994 (Japan)

**TABLE 2:** Active clinical trials in nanomedicine for cancer treatment.^166^

NCT number	Study title	Study status	Conditions	Sponsor	Phases
NCT00609791	Paclitaxel albumin‐stabilized nanoparticle formulation in treating patients of different ages with metastatic breast cancer	Active not recruiting	Breast cancer	City of Hope Medical Center	Phase 2
NCT01463072	Nab‐paclitaxel in treating older patients with locally advanced or metastatic breast cancer	Active not recruiting	Locally advanced breast carcinoma, metastatic breast carcinoma, recurrent breast carcinoma, stage III breast cancer AJCC, stage IIIA breast cancer AJCC, stage IIIB breast cancer AJCC, stage IIIC breast cancer AJCC, stage IV breast cancer AJCC	City of Hope Medical Center	Phase 2
NCT01525966	Carboplatin and paclitaxel albumin‐stabilized nanoparticle formulation before surgery in treating patients with locally advanced or inflammatory triple negative breast cancer	Active not recruiting	Inflammatory breast cancer, stage IIA breast cancer, stage IIIA breast cancer, stage IIIB breast cancer, stage IIIC breast cancer, triple‐negative breast cancer, stage IIB breast cancer, estrogen receptor negative, progesterone receptor negative, HER2/Neu negative	City of Hope Medical Center	Phase 2
NCT01730833	Pertuzumab, trastuzumab, and paclitaxel albumin‐stabilized nanoparticle formulation in treating patients with HER2‐positive advanced breast cancer	Active not recruiting	HER2‐positive breast cancer, recurrent breast cancer, stage IIA breast cancer, stage IIB breast cancer, stage IIIA breast cancer, stage IIIB breast cancer, stage IIIC breast cancer, stage IV breast cancer, breast adenocarcinoma, inflammatory breast carcinoma	City of Hope Medical Center	Phase 2
NCT01847326	Paclitaxel albumin‐stabilized nanoparticle formulation and carboplatin followed by chemoradiation in treating patients with recurrent head and neck cancer	Active not recruiting	Recurrent salivary gland cancer, recurrent squamous cell carcinoma of the hypopharynx, recurrent squamous cell carcinoma of the larynx, recurrent squamous cell carcinoma of the lip and oral cavity, recurrent squamous cell carcinoma of the nasopharynx, recurrent squamous cell carcinoma of the oropharynx, recurrent squamous cell carcinoma of the paranasal sinus and nasal cavity, recurrent verrucous carcinoma of the larynx, recurrent verrucous carcinoma of the oral cavity, salivary gland squamous cell carcinoma, tongue cancer	University of Chicago	Phase 1
NCT02020707	Nab‐paclitaxel and bevacizumab in treating patients with unresectable stage IV melanoma or gynecological cancers	Active not recruiting	Cervical adenocarcinoma, cervical adenosarcoma, cervical adenosquamous carcinoma, cervical carcinosarcoma, cervical squamous cell carcinoma, clinical stage IV cutaneous melanoma AJCC, endometrial adenosquamous carcinoma, endometrial clear cell adenocarcinoma, endometrial endometrioid adenocarcinoma, endometrial mixed cell adenocarcinoma, endometrial mucinous adenocarcinoma, endometrial serous adenocarcinoma, endometrial undifferentiated carcinoma, fallopian tube adenocarcinoma, fallopian tube carcinosarcoma, fallopian tube clear cell adenocarcinoma, fallopian tube endometrioid adenocarcinoma, fallopian tube mucinous adenocarcinoma, fallopian tube serous adenocarcinoma, fallopian tube squamous cell carcinoma, fallopian tube transitional cell carcinoma, fallopian tube undifferentiated carcinoma, malignant peritoneal neoplasm, ovarian carcinosarcoma, ovarian clear cell adenocarcinoma, ovarian endometrioid adenocarcinoma, ovarian high grade serous adenocarcinoma, ovarian mucinous adenocarcinoma, ovarian serous adenocarcinoma, ovarian transitional cell carcinoma, ovarian undifferentiated carcinoma, primary peritoneal Carcinosarcoma, Primary Peritoneal Clear Cell Adenocarcinoma, Primary Peritoneal Serous Adenocarcinoma, Primary Peritoneal Transitional Cell Carcinoma, Primary Peritoneal Undifferentiated Carcinoma, Unresectable Melanoma, Uterine Corpus Carcinosarcoma	Mayo Clinic	Phase 1
NCT02106598	Targeted silica nanoparticles for real‐time image‐guided intraoperative mapping of nodal metastases	Active not recruiting	Head and neck melanoma	Memorial Sloan Kettering Cancer Center	Phase 1, Phase 2
NCT02258659	Nab‐paclitaxel and carboplatin followed by response‐based local therapy in treating patients with stage III or IV HPV‐related oropharyngeal cancer	Active not recruiting	Human papilloma virus infection, stage III squamous cell carcinoma of the oropharynx, stage IVA squamous cell carcinoma of the oropharynx, stage IVB squamous cell carcinoma of the oropharynx	University of Chicago	Phase 2
NCT02336087	Gemcitabine hydrochloride, paclitaxel albumin‐stabilized nanoparticle formulation, metformin hydrochloride, and a standardized dietary supplement in treating patients with pancreatic cancer that cannot be removed by surgery	Active not recruiting	Pancreatic adenocarcinoma, unresectable pancreatic carcinoma, stage III pancreatic cancer AJCC and, stage IV pancreatic cancer AJCC	City of Hope Medical Center	Phase 1
NCT02530489	Nab‐paclitaxel and atezolizumab before surgery in treating patients with triple negative breast cancer	Active not recruiting	Breast adenocarcinoma, invasive breast carcinoma, triple‐negative breast carcinoma	M.D. Anderson Cancer Center	Phase 2
NCT02631733	Liposomal irinotecan and veliparib in treating patients with solid tumors	Active not recruiting	Malignant solid neoplasm	National Cancer Institute (NCI)	Phase 1
NCT02716012	First‐in‐human safety, tolerability and antitumour activity study of MTL‐CEBPA in patients with advanced liver cancer	Active not recruiting	Hepatocellular carcinoma, liver cancer	Mina Alpha Limited	Phase 1
NCT02769962	Trial of EP0057, a nanoparticle camptothecin with olaparib in people with relapsed/refractory small cell lung cancer	Recruiting	Urothelial carcinoma, urothelial cancer, lung neoplasms, small cell lung cancer, prostate cancer	National Cancer Institute (NCI)	Phase 1, Phase 2
NCT02975882	Nanoparticle albumin‐bound rapamycin, temozolomide, and irinotecan hydrochloride in treating pediatric patients with recurrent or refractory solid tumors	Active not recruiting	Childhood solid neoplasm, recurrent malignant solid neoplasm, recurrent primary central nervous system neoplasm, refractory malignant solid neoplasm, refractory primary central nervous system neoplasm	Children's Oncology Group	Phase 1
NCT03308604	AGuIX gadolinium‐based nanoparticles in combination with chemoradiation and brachytherapy	Recruiting	Gynecologic cancer	Gustave Roussy, Cancer Campus, Grand Paris	Phase 1
NCT03337087	Liposomal irinotecan, fluorouracil, leucovorin calcium, and rucaparib in treating patients with metastatic pancreatic, colorectal, gastroesophageal, or biliary cancer	Active not recruiting	Metastatic biliary tract carcinoma, metastatic colorectal carcinoma, metastatic gastroesophageal junction adenocarcinoma, metastatic malignant digestive system neoplasm, metastatic pancreatic adenocarcinoma, stage IV colorectal cancer AJCC, stage IV pancreatic cancer AJCC and, stage IVA colorectal cancer AJCC, stage IVB colorectal cancer AJCC	Academic and Community Cancer Research United	Phase 1, Phase 2
NCT03606967	Testing the addition of an individualized vaccine to Nab‐paclitaxel, durvalumab and tremelimumab and chemotherapy in patients with metastatic triple negative breast cancer	Recruiting	Anatomic stage IV breast cancer AJCC, invasive breast carcinoma, metastatic triple‐negative breast carcinoma	National Cancer Institute (NCI)	Phase 2
NCT03656835	Nanochip technology in monitoring treatment response and detecting relapse in participants with diffuse large B‐cell lymphoma	Recruiting	Diffuse large B‐cell lymphoma, diffuse large B‐cell lymphoma germinal center B‐cell type, diffuse large B‐cell lymphoma, not otherwise specified, high grade B‐cell lymphoma	Ohio State University Comprehensive Cancer Center	NA
NCT03660930	Nab‐sirolimus and pazopanib hydrochloride in treating patients with advanced nonadipocytic soft tissue sarcomas	Active not recruiting	Advanced soft tissue sarcoma, locally advanced soft tissue sarcoma, metastatic soft tissue sarcoma	University of Washington	Phase 1, Phase 2
NCT03736720	Liposomal irinotecan, fluorouracil and leucovorin in treating patients with refractory advanced high grade neuroendocrine cancer of gastrointestinal, unknown, or pancreatic origin	Active not recruiting	Locally advanced digestive system neuroendocrine carcinoma, locally advanced pancreatic neuroendocrine carcinoma, metastatic digestive system neuroendocrine carcinoma, metastatic pancreatic neuroendocrine carcinoma, refractory digestive system neuroendocrine carcinoma, refractory pancreatic neuroendocrine carcinoma, unresectable digestive system neuroendocrine carcinoma, unresectable pancreatic neuroendocrine carcinoma	Roswell Park Cancer Institute	Phase 2
NCT03915444	Nab‐paclitaxel + cisplatin + gemcitabine in untreated metastatic pancreatic adenocarcinoma	Active not recruiting	Pancreatic ductal adenocarcinoma	HonorHealth Research Institute	Phase 2
NCT03961698	Evaluation of IPI‐549 combined with front‐line treatments in pts. with triple‐negative breast cancer or renal cell carcinoma	Active not recruiting	Breast cancer, renal cell carcinoma	Infinity Pharmaceuticals, Inc.	Phase 2
NCT04033354	A randomized, double‐blind, placebo controlled phase III study to investigate efficacy and safety of first‐line treatment with HLX10 + chemotherapy (carboplatin‐nanoparticle albumin bound (Nab) paclitaxel) in patients with stage IIIB/IIIC or IV NSCLC	Active not recruiting	Squamous non‐small cell lung cancer	Shanghai Henlius Biotech	Phase 3
NCT04115163	Biologically optimized infusion schedule of gemcitabine and Nab‐paclitaxel for the treatment of metastatic pancreatic cancer	Recruiting	Metastatic pancreatic adenocarcinoma, stage IV pancreatic cancer AJCC	Anne Noonan	Phase 2
NCT04137653	Treatment of triple‐negative breast cancer with albumin‐bound paclitaxel as neoadjuvant therapy: a prospective RCT	Recruiting	Breast cancer	Shengjing Hospital	Phase 3
NCT04158635	Gemcitabine, Nab‐paclitaxel, and bosentan for the treatment of unresectable pancreatic cancer	Recruiting	Stage III pancreatic cancer AJCC, stage IV pancreatic cancer AJCC, unresectable pancreatic carcinoma	City of Hope Medical Center	Phase 1
NCT04167969	The use of nanoparticles to guide the surgical treatment of prostate cancer	Recruiting	Prostate cancer	Memorial Sloan Kettering Cancer Center	Phase 1
NCT04216472	Nab‐paclitaxel and alpelisib for the treatment of anthracycline refractory triple negative breast cancer with PIK3CA or PTEN alterations	Active not recruiting	Anatomic stage I breast cancer AJCC, anatomic stage IA breast cancer AJCC, anatomic stage IB breast cancer AJCC, anatomic stage ii breast cancer AJCC, anatomic stage IIA breast cancer AJCC, anatomic stage IIB breast cancer AJCC, anatomic stage III breast cancer AJCC, anatomic stage IIIA breast cancer AJCC, anatomic stage IIIB breast cancer AJCC, anatomic stage IIIC breast cancer AJCC, prognostic stage I breast cancer AJCC, prognostic stage IA breast cancer AJCC, prognostic stage IB breast cancer AJCC, prognostic stage II breast cancer AJCC, prognostic stage IIA breast cancer AJCC, prognostic stage IIB breast cancer AJCC, prognostic stage III breast cancer AJCC, prognostic stage IIIA breast cancer AJCC, prognostic stage IIIB breast cancer AJCC, prognostic stage IIIC breast cancer AJCC, refractory breast carcinoma, triple‐negative breast carcinoma	M.D. Anderson Cancer Center	Phase 2
NCT04233866	Comparing two treatment combinations, gemcitabine and Nab‐paclitaxel with 5‐fluorouracil, leucovorin, and liposomal irinotecan for older patients with pancreatic cancer that has spread	Active not recruiting	Metastatic pancreatic adenocarcinoma, stage IV pancreatic cancer AJCC	ECOG‐ACRIN Cancer Research Group	Phase 2
NCT04240639	An extension study MRI/US fusion imaging and biopsy in combination with nanoparticle directed focal therapy for ablation of prostate tissue	Active not recruiting	Neoplasms of the prostate	Nanospectra Biosciences, Inc.	NA
NCT04390958	Neoadjuvant chemotherapy With Nab‐paclitaxel plus cisplatin and capecitabine for locally advanced thoracic esophageal squamous cell carcinoma	Recruiting	Esophageal squamous cell carcinoma	Chinese Academy of Medical Sciences	Phase 2
NCT04524702	Paricalcitol and hydroxychloroquine in combination with gemcitabine and Nab‐paclitaxel for advanced pancreatic cancer	Active not recruiting	Advanced pancreatic adenocarcinoma, metastatic pancreatic adenocarcinoma, stage IV pancreatic cancer AJCC	Emory University	Phase 2
NCT04645147	Safety and immunogenicity of an Epstein–Barr virus (EBV) gp350‐ferritin nanoparticle vaccine in healthy adults with or without EBV infection	Recruiting	EBV, Epstein–Barr virus infection, infectious mononucleosis	National Institute of Allergy and Infectious Diseases (NIAID)	Phase 1
NCT04682847	Radiotherapy with iron oxide nanoparticles (SPION) on MR‐Linac for primary and metastatic hepatic cancers	Active not recruiting	Liver neoplasms, hepatic cirrhosis, hepatic carcinoma, liver cancer, liver metastases, liver carcinoma, hepatocellular carcinoma, hepatocellular cancer, hepatic atrophy	Allegheny Singer Research Institute (also known as Allegheny Health Network Research Institute)	
NCT04734262	A phase II study to explore the safety, tolerability, and preliminary antitumor activity of sitravatinib plus tislelizumab or combination with Nab‐paclitaxel in patients with locally recurrent or metastatic triple negative breast cancer (TNBC)	Active not recruiting	Metastatic breast cancer	Fudan University	Phase 2
NCT04751786	Dose escalation study of immunomodulatory nanoparticles	Recruiting	Advanced solid tumor	Radboud University Medical Center	Phase 1
NCT04784221	Nanoparticles and hypofractionated protontherapy for reirradiation of pantumor relapse	Not yet recruiting	Recurrent cancer, previous radiation	Centre Francois Baclesse	Phase 2
NCT04789486	Nano‐SMART: nanoparticles with MR guided SBRT in centrally located lung tumors and pancreatic cancer	Recruiting	Non‐small cell lung cancer, advanced pancreatic adenocarcinoma, unresectable pancreatic cancer, ductal adenocarcinoma of the pancreas	Dana‐Farber Cancer Institute	Phase 1, Phase 2
NCT04808531	NanaBis, an oro‐buccal administered delta9‐tetrahydrocannabinol (d9‐THC) & cannabidiol (CBD) medicine for the management of bone pain from metastatic cancers	Not yet recruiting	Cancer‐related pain	Medlab Clinical	Phase 3
NCT04881032	AGuIX nanoparticles with radiotherapy plus concomitant temozolomide in the treatment of newly diagnosed glioblastoma	Recruiting	Glioblastoma	Centre Jean Perrin	Phase 1, Phase 2
NCT04899908	Stereotactic brain‐directed radiation with or without aguix gadolinium‐based nanoparticles in brain metastases	Recruiting	Brain cancer, brain metastases, melanoma, lung cancer, breast cancer, HER2‐positive breast cancer, colorectal cancer, gastrointestinal cancer, SRS, SRT, whole brain radiation, stereotactic radiation, AGuIX, nanoparticle, cystic, brain tumor	Dana‐Farber Cancer Institute	Phase 2
NCT05101616	A pilot study of neoadjuvant chemotherapy with or without camrelizumab for locally advanced gastric cancer	Recruiting	Gastric cancer	Shanghai Minimally Invasive Surgery Center	Phase 1, Phase 2
NCT05103358	Phase 2 basket trial of Nab‐sirolimus in patients with malignant solid tumors with pathogenic alterations in TSC1/TSC2 genes (PRECISION 1)	Recruiting	Tumor, tumor, solid, metastasis, metastatic cancer, cancer, cancer metastatic, tumors, neoplasms, neoplasm metastasis, solid tumor, advanced solid tumor, advanced cancer, malignant solid tumor, malignant solid neoplasm, malignant neoplasm, malignant tumor, TSC, TSC1, TSC2, metastatic solid tumor, metastatic neoplasm	Aadi Bioscience, Inc.	Phase 2
NCT05264974	Novel RNA‐nanoparticle vaccine for the treatment of early melanoma recurrence following adjuvant anti‐PD‐1 antibody therapy	Recruiting	Melanoma	University of Florida	Phase 1
NCT05285358	Pressurized intraperitoneal aerosolized nab‐paclitaxel in combination with gemcitabine and cisplatin for the treatment of biliary tract cancer patients with peritoneal metastases	Recruiting	Distal bile duct adenocarcinoma, gallbladder carcinoma, intrahepatic cholangiocarcinoma, metastatic malignant neoplasm in the peritoneum, stage IV distal bile duct cancer AJCC, stage IV intrahepatic bile duct cancer AJCC, stage IV intrahepatic cholangiocarcinoma AJCC, stage IVB gallbladder cancer AJCC	City of Hope Medical Center	Phase 1
NCT05359783	Sentinel node localization and staging with low dose superparamagnetic iron oxide	Active not recruiting	Breast cancer, sentinel lymph node	Sahlgrenska University Hospital, Sweden	Phase 1, Phase 2
NCT05422794	Testing the addition of anti‐cancer drug, ZEN003694 (ZEN‐3694) and PD‐1 inhibitor (pembrolizumab), to standard chemotherapy (Nab‐paclitaxel) treatment in patients with advanced triple‐negative breast cancer	Recruiting	Anatomic stage III breast cancer AJCC, anatomic stage IV breast cancer AJCC, locally advanced triple‐negative breast carcinoma, metastatic triple‐negative breast carcinoma, unresectable triple‐negative breast carcinoma	National Cancer Institute (NCI)	Phase 1
NCT05456022	Therapeutic efficacy of quercetin versus its encapsulated nanoparticle on tongue squamous cell carcinoma cell line	Not yet recruiting	Oral cancer	Cairo University	Phase 2
NCT05816694	The efficacy and safety of nanoparticle albumin‐bound (NAB)‐paclitaxel plus cisolation versus CEP (cisplatin, epirubicin, cyclophosphamide) in induction therapy for thymoma: a study for a single‐center prospective phase II randomized controlled train	Not yet recruiting	Histological or cytological confirmed thymoma	Peng Liu	Phase 2
NCT06048367	Carbon nanoparticle‐loaded iron [CNSI‐Fe(II)] in the treatment of advanced solid tumor	Recruiting	Advanced solid tumor, lung cancer, pancreas cancer, breast cancer, thyroid cancer, colorectal cancer, cervical cancer, ovarian cancer, vulva cancer	Sichuan Enray Pharmaceutical Sciences Company	Phase 1
NCT06173349	PLZ4‐coated paclitaxel‐loaded micelles for the treatment of patients with recurrent or refractory non‐muscle invasive bladder cancer	Recruiting	Recurrent non‐muscle invasive bladder carcinoma, stage 0a bladder cancer AJCC, stage 0is bladder cancer AJCC, stage I bladder cancer AJCC	Mamta Parikh	Phase 1

## 7 PERSPECTIVES AND CHALLENGES

While the field is rapidly evolving, here are some perspectives for future advancements of personalized nanotherapy in oncology and issues to be addressed to pave the way for use in clinical practice.

### 7.1 Main prospects for the evolution of nano‐based precision oncology

- *Nano immune‐engineering*. Cancer immunotherapy has demonstrated great clinical benefit leading to a durable response in different types of cancers in advanced stages. However, off‐target effects, systemic toxicity, and the development of treatment resistance are limits that need to be overcome. Nanoscale immune‐engineering is being developed to achieve, by fine tuning the properties of the nanomaterials, such as size, shape, charge, and surface chemistry, tumor/immune cell‐specific targeting and engineering, and to enhance both the efficacy and safety, maximizing bioactivity and bioavailability of current immunotherapies, such as CAR‐T therapy^210^ and checkpoint inhibitors.^211^ CAR‐T therapy has resulted in a high remission rate in patients with B‐cell malignancies^212^ but suffers from a complex manufacturing process and a limited success in solid tumors (due to the paucity of tumor‐specific antigens and the immunosuppressive TME).^213^ Nanocarriers can drive cell selective delivery of CAR‐T therapy and overcome the existing challenges. Monoclonal Ab‐based checkpoint blockade therapy has remarkable clinical success in different solid tumors, but premature degradation of the systemically administered Abs and off‐target toxicities lower its efficacy.^214^ Nanocarriers can drive immune‐cell selective delivery of CAR‐T therapy, as well as tumor specific delivery of anti‐PD‐1 Abs and may enable combined treatments to overcome the existing challenges.^211^
- *Multimodal nanoplatforms*. The future of cancer nanomedicine rests on multimodal nanoplatforms that combine targeting ligands, imaging and diagnostic agents, and therapeutic components into a single unit of functionalized nanoparticles. Thus, multifunctionality is a powerful and unique advantage of nanotheranostic over traditional methods, and evidence has shown its capacity to work efficiently and noninvasively in vivo without systemic toxicity.^53^ Nanotheranostic not only provides the means for early diagnostic tools,^215^ nanoimaging‐therapeutic integrated medicine,^216^ targeted‐therapy,^217^ and tumor‐specific nanodelivery agent,^218^ it also holds potential for real‐time monitoring of drug response and a reduction of the side effects and drug toxicity in patients.^52^ By combining therapeutic functionalities with molecular imaging, theranostic‐based strategies may be beneficial in the selection of therapy, planning of treatment, monitoring of objective response and planning of follow‐up therapy based on the specific molecular characteristics of a cancer.

### 7.2 Main challenges to overcome

*Design and production of patient‐specific nanoparticles*. Advances in nanotechnology may allow the customization of nanotherapy based on the patient's genetic, molecular, and immunological profile. This could lead to highly personalized treatment strategies tailored to individual cancer characteristics.

- *Data‐driven decision making*. Integration of nanomedicine with AI and data analytics can enhance the interpretation of complex molecular data. This could contribute to more accurate diagnostics, treatment predictions, and personalized medicine strategies.
- *Interdisciplinary research by cross‐disciplinary teams*. Advances in nanomedicine for personalized oncology will require collaboration between researchers, clinicians, engineers, and data scientists to integrate different expertise and skills and thus accelerate progress.
- *Safety and health concerns*. There are ongoing debates about the potential short‐ and long‐term health impacts of nanoparticles developed for diagnostic, drug delivery, or theragnostic purposes.^219^ Their physicochemical characteristics dramatically influence the biodistribution, metabolism, routes of clearance, as well as biocompatibility and, at the subcellular level, their interactions with oxidative organelles, such as mitochondria, with redox active proteins, and the activation of different signaling routes via interaction with cell surface receptors.^220^, ^221^
- *Regulation*. Nanotechnology presents regulatory challenges due to its novelty and the complexity of assessing its risks. Governments and regulatory bodies face difficulties in establishing appropriate guidelines and standards for the safe use of nanomaterials.^222^ The regulatory landscape is sketchy with very few guidance and consensus definitions,^223^ although a new guidance was recently issued by the US FDA.^224^ To establish the risk‐benefit balance for marketing authorization, the main issues to be addressed are those related to pharmaceutical quality, efficacy, and nonclinical and clinical safety. Subsequently, relevant vigilance tools for a continuous risk‐benefit assessment approach are needed.
- *Ethical and social implications*, which include, (a) *Privacy and genetic information*. Nanomedicine often involves the collection and analysis of genetic data. This raises privacy concerns regarding the storage, usage, and protection of sensitive genetic information.^225^ (b) *The long‐term effects and unknown risks of nanomedicine*, which are not always fully understood, particularly in the context of oncology where patients may already be undergoing aggressive therapies. There's a need for ongoing monitoring and research to assess the potential risks and benefits over time. (c) *Issues concerning intellectual property rights and commercialization*, which can influence the accessibility and affordability of nanomedicine treatments. Patent protections may limit competition and drive‐up prices, making these treatments inaccessible for public health.^226^
- *Scale‐up challenges*. While nanoscale research often yields promising results at the laboratory scale, transitioning to large‐scale production can be difficult. Maintaining the desired properties and performance of nanomaterials at scale while keeping costs manageable is a significant challenge.^227^
- *Affordability and accessibility*. Price poses an important challenge for companies producing nanotherapeutics. Nanodrugs are significantly more expensive to manufacture than conventional medicines,^228^, ^229^ leading to significantly higher selling prices and acquisition costs for hospitals, hindering the entry of nanomedicine into routine clinical practice. Anticancer agents represent the most advanced and largest niche of the nanomedicine market, accounting for almost 50% of revenue, and it is expected to expand in the future, given the growing number of next‐generation nanodrugs undergoing clinical investigation.^230^ Efforts should be made to develop cost‐effective nanomedicine solutions to ensure broader accessibility, especially in resource‐limited settings. The prospect of reducing healthcare costs, due to the reduced risk of side effects typical of nanomedicine, and the reduced dosage and greater effectiveness of drugs, due to their targeted and monitored distribution, must guide public and private reversals in the personalized nanomedicine sector. The development of nanotechnology aimed at precision oncology is essential for the future of healthcare.

## 8 CONCLUSIONS

Nanomedicine has the potential to revolutionize cancer care by offering patient‐centered approaches that (a) improve the sensitivity of imaging techniques, allowing for earlier detection of cancer, (b) allow for the customization of treatment based on the patient's specific cancer type, immunological, and genetic profiles, and (c) enable the targeted delivery of drugs, which enhance efficacy and tolerability while reducing systemic toxicity of the treatment, providing real‐time monitoring of the patient's response to therapy for dynamic adjustments of the therapeutic plan.

By minimizing side effects, reducing the need for hospitalization, and allowing for outpatient treatments, nanomedicine may enhance the overall patient experience and improve quality of life. With the integration of nanomedicine into personalized treatment plans, patients can be more actively involved in their care decisions. This empowerment aligns with the principles of patient‐centered care, where the patient's preferences, needs, and values guide clinical decisions. However, to fully realize its potential, ongoing research, patient education, and ethical considerations must be addressed to ensure that nanomedicine is accessible, safe, and beneficial for all cancer patients.

9

*CRISPR/Cas9 technology*: a technology that enables the editing of parts of the genome by removing, adding, or altering sections of the DNA sequence. The CRISPR/Cas9 system consists of two key molecules:

- an enzyme called Cas9, that can cut the two strands of DNA at a specific location in the genome, so that sequences of DNA can then be added or removed.
- an RNA sequence, called guide RNA (gRNA), that guides Cas9 to the selected part of the genome.

*Immunogram*: a graphical representation of the parameters that characterize the interaction between cancer and immune system, in a specific patient (e.g., presence of immune‐effector or immune‐suppressor cells in the tumor microenvironment, expression of immune exhaustion markers or immune checkpoint molecules), which can help guide treatment decisions.

*Immunoliposomes*: liposomes conjugated with monoclonal Abs, which specifically recognize and bind to the surface antigens of specific cells, ensuring that liposomes and the conveyed drug, can selectively reach and accumulate in the target organs or tissues.

*Liquid biopsy*: the sampling and analysis of body fluid, such as blood, urine, or saliva, to detects circulating tumor DNA (ctDNA), CTCs, exosomes, or other molecular markers.

*Microfluidics*: the technology of manipulating and controlling fluids at a very small scale, typically in channels with dimensions ranging from tens to hundreds of micrometers.

*Nanobiosensor*: an analytical device, in nanometer scales, which interacts with the targeted analyte and produces a measurable physical signal, allowing accurate analysis of physiological and pathological processes in living cells.

*Nanofiber*: a fiber with a cross‐sectional diameter that ranges from 10 to 100 nm.

*Nanomedicine*: the medical application of nanotechnology which employs nanoscale materials (measuring between 1 and 100 nm) and devices for diagnosis, treatment, and prevention of diseases.

*Nanotechnology*: the branch of technology that deals with dimensions ranging 1–100 nm.

*Nanotube*: a nanoscale material that has a tube‐like structure, typically measuring 3−30 nm in outer diameter.

*Organ‐on‐a‐chip*: an integrated circuit containing a 3D microfluidic cell culture, that simulates the activities, mechanics, and physiological response of an entire organ or an organ system.

*Precision oncology*: a personalized approach to cancer treatment that, unlike traditional cancer treatments that follow a one‐size‐fits‐all model, tailors therapy based on the individual genetic and molecular profile of a patient's tumor.

*Targeted therapy*: a type of cancer treatment that precisely targets specific molecules or genetic alterations that drive the growth, progression or spread of cancer and that focuses on cancer cells, while sparing normal cells, often result in fewer side effects.

## AUTHOR CONTRIBUTIONS

*Writing—original draft, writing—review and editing*: Emma Di Carlo. *Data curation, writing—review and editing*: Carlo Sorrentino. *Investigation, methodology*: Stefania Livia Ciummo and Cristiano Fieni. All authors have read and approved the final manuscript.

## CONFLICT OF INTEREST STATEMENT

The authors declare no conflict of interest.

## ETHICS STATEMENT

Not applicable.

## Data Availability

Data sharing not applicable to this article as no datasets were generated or analyzed during the current study.
